# Hyperspectral Multiplexed Biological Imaging of Nanoprobes Emitting in the Short-Wave Infrared Region

**DOI:** 10.1186/s11671-019-3068-x

**Published:** 2019-07-19

**Authors:** A. Yakovliev, R. Ziniuk, D. Wang, B. Xue, L. O. Vretik, O. A. Nikolaeva, M. Tan, G. Chen, Yu. L. Slominskii, J. Qu, T. Y. Ohulchanskyy

**Affiliations:** 1 Key Laboratory of Optoelectronic Devices and Systems of Ministry of Education and Guangdong Province, College of Physics and Optoelectronic Engineering, Shenzhen University, Shenzhen, Guangdong Province, 518060 People’s Republic of China; 2 Taras Shevchenko National University of Kyiv, Kyiv, 01601 Ukraine; 3 School of Chemistry and Chemical Engineering, Harbin Institute of Technology, Harbin, 150001 People’s Republic of China; 4 Institute of Organic Chemistry NASU, Kyiv, 02094 Ukraine

**Keywords:** Hyperspectral imaging, Photoluminescence, Fluorescence bioimaging, Multiplexed nanoprobes, Short wavelength infrared

## Abstract

Optical bioimaging with exogenous luminophores emitting in short-wave infrared spectral region (SWIR, ~ 1000–1700 nm) is a rapidly developing field, and the development of multiple SWIR-photoluminescent nanoprobes has recently been reported. In this regard, hyperspectral imaging (HSI), combined with unmixing algorithms, is a promising tool that can allow for efficient multiplexing of the SWIR-emitting nanoagents by their photoluminescence (PL) spectral profiles. The SWIR HSI technique reported here is developed to multiplex two types of nanoprobes: polymeric nanoparticles doped with organic dye (PNPs) and rare-earth doped fluoride nanoparticles (RENPs). Both types of nanoprobes exhibit PL in the same spectral range (~ 900–1200 nm), which hinders spectral separation of PL with optical filters and limits possibilities for their multiplexed imaging in biological tissues. By applying SWIR HSI, we exploited differences in the PL spectral profiles and achieved the spectrally selective and sensitive imaging of the PL signal from every type of nanoparticles. Unmixing of acquired data allowed for multiplexing of the spectrally overlapping nanoprobes by their PL profile. Both quantitative and spatial distribution for every type of nanoparticles were obtained from their mixed suspensions. Finally, the SWIR HSI technique with unmixing protocol was applied to in vivo imaging of mice subcutaneously injected with PNPs and RENPs. The applicability of hyperspectral techniques to multiplex nanoprobes in the in vivo imaging was successfully demonstrated.

## Introduction

Biomedical imaging technologies are rapidly developing in recent decades, allowing for early detection and assessment of various diseases and pathologies. Among different imaging modalities, optical imaging holds a unique position due to its high spatial and temporal resolution and relatively low cost. Multiple optical imaging approaches based on photoluminescence (or, more specifically, fluorescence) are under development and being clinically translated. For example, lymphatic system imaging and intraoperative fluorescence imaging-guided surgery have shown promising results to advance healthcare [1, 2]. On the other hand, exogenous photoluminescent probes targeting specific regions of interest (e.g., tumor) are actively developed for in vivo and ex vivo imaging. In addition to the usual requirements for photoluminescence (PL) probes (i.e., high absorbance, emission quantum yield, and photostability), spectral position and shape of PL emission are also vital parameters to be considered. As the attenuation of light by biological tissues is known to be lower in the near-infrared (NIR) spectral range (~ 700–950 nm) than in the visible one, an existence of the NIR transparency window for biological tissues (~ 700–950 nm) has been introduced and a lot of efforts are devoted to the development and applications of the NIR emitting probes [3–6]. Moreover, recent advances led to the introduction of the second and third NIR windows (NIR-II and NIR-III) in the spectral range of ~ 1000–1700 nm, which is often termed as short-wave infrared (SWIR), especially by manufacturers in the rapidly developing field of infrared imaging [7–9]. Despite higher water absorption in SWIR range in a comparison to the conventional NIR window, lower autofluorescence and scattering of biological tissues allow for superior imaging resolution and higher imaging depth in the SWIR PL bioimaging [10–12]. For instance, in SWIR PL imaging of lymphatic and brain vasculature, using novel SWIR fluorescent dye CH1055-PEG, resolution and signal to background ratio were shown to be superior in a comparison with conventional NIR PL imaging with NIR-I fluorescent dye, indocyanine green [13]. Moreover, use of the SWIR-emitting nanoprobes (single-walled carbon nanotubes) and SWIR imaging camera allowed Dai’s group to visualize sub-10 μm vessels at the depth of > 2 mm in non-invasive (without craniotomy) imaging of mice cerebrovasculature, which is inaccessible for PL imaging in visible or NIR-I ranges [8].

Organic dyes and dye complexes with intense absorption in the first NIR window and fluorescence in NIR-II window could be considered as promising NIR-SWIR probes; they were shown to serve as an exceptional contrast agent for vasculature and lymph node imaging, tumor delineation, and image-guided surgery [13–16]. It is worth noting that the organic dye indocyanine green (ICG) is the only NIR fluorescence contrast agent currently approved by the US Food and Drugs Administration for use in humans [17]. At the same time, molecular imaging probes (i.e., dyes or dye complexes) have limitations associated with necessity to modify their molecular structure for changing their bioprobe characteristics (e.g., water solubility, cell permeability, etc.) or furnishing them with other imaging or targeting modalities. In contrast, nanoparticles (NPs) comprising PL centers can have their surface been covalently modified with different moieties for improved water dispersibility and stability, controlled surface charge, or targeting purposes. In addition, introduction of NIR-SWIR PL nanoplatforms allows for combination of PL imaging with other imaging, diagnostic, or therapeutic modalities. Recent studies report deep tissue, whole body, tumor, or transcranial imaging with SWIR-emitting nanoformulations used for monitoring various processes in vivo [14, 18–21]. Among various reported NIR-SWIR-emitting nanoprobes for in vivo imaging, two types can be distinguished: with NIR-SWIR fluorescence arising from organic moieties (i.e., conjugated polymer) or ceramic (e.g., fluoride) nanocrystals doped with rare-earth ions. Polymer-based nanoparticles (PNPs) are among the most successful nanomedicines in clinical translations, due to relative ease in synthesis and chemical functionalization, as well as superior biocompatibility and biodegradability [22]. When loaded with NIR-SWIR fluorophores, PNPs can serve as promising imaging probes or imaging-guided drug delivery vehicles [23, 24]. On the other hand, rare-earth ion-doped nanoparticles (RENPs) are a well-known class of nanoprobes, which have unique photoluminescence properties accessible through both upconversion (anti-Stokes-shifted) and down-conversion (Stokes-shifted) processes [25–30]. Recently, RENPs have been translated for use in NIR-SWIR imaging. In contrast to organic moieties based NIR-SWIR probes, they possess high quantum yield, exceptional photostability, and narrow emission bands in whole NIR-SWIR spectral region, which can be tuned through doping with various ions [20, 31, 32]. RENPs were applied to small animal vasculature and organs imaging, tumor detection, multiplexed, and multispectral imaging [3, 19, 20, 33–35].

With emerging development of PL bioimaging, an ability to simultaneously track several PL moieties in vivo can be required for different purposes (e.g., targeted imaging of the selected cells or organs along with imaging-guided drug delivery). To address this challenge, multiplexed imaging methods were developed. Multiplexed imaging refers to the complementarity of anatomical and functional information in the imaged biological system; its application can allow for the combination of imaging biomarkers, contrasts, and modalities to increase the utility of imaging in research and clinic applications [36]. Multiplexed PL imaging can enhance theranostic dimension of nanomedicine, offering the ability to introduce several PL imaging contrasts along with therapeutic modality. Most commonly used multiplexed imaging methods distinguish PL probes by spectral position of their PL emission, using appropriate optical filters [37–39]. However, proper multiplexing in such an approach requires utilization of nanoprobes with spectrally narrow, non-overlapping PL spectra. In this regard, hyperspectral imaging (HSI) combined with spectral mixture analysis algorithms is a promising tool for PL multiplexing. However, biomedical applications of PL HSI are mostly limited to fluorescence microscopy, for multiplexing different types of nanoprobes and eliminating background and autofluorescence [40, 41]. In regard to in vivo HSI, it is most frequently used in reflection-imaging mode through acquisition and sequential analysis of the tissue reflection spectra [42], though HSI imaging in vivo (also called multispectral imaging) has also been reported for PL in visible and NIR ranges [3, 5]. However, no reports on HSI of SWIR-emitting nanoprobes could be found in the literature.

**Fig. 1:**
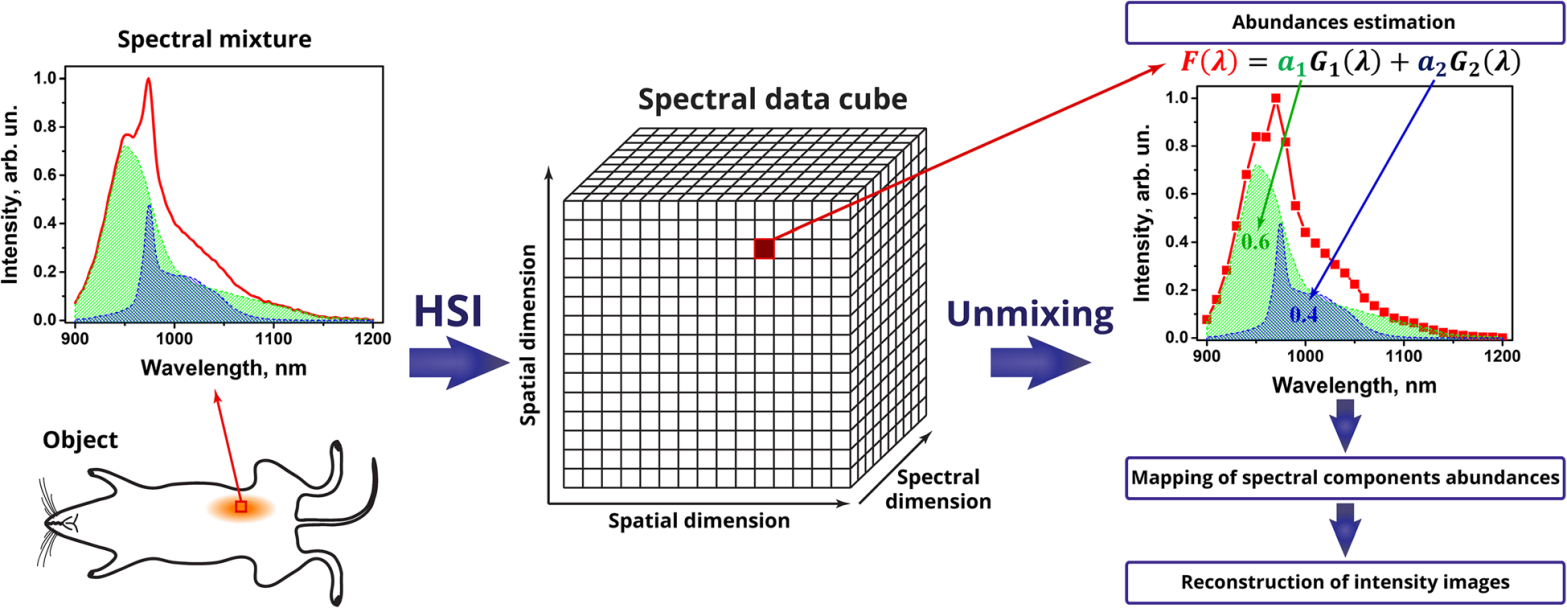
Scheme illustrating application of HSI for multiplexing photoluminescent nanoprobes

Recently, we have reported the development of band sequential HSI system combined with spectral unmixing software for SWIR PL imaging [43]. Band sequential HSI procedure was based on consecutive acquisition of 2D images through an element with spectrally varied transmittance (i.e., liquid crystal tunable filter, LCTF). The SWIR PL data obtained from RENPs suspensions were presented as three-dimensional spectral data cube (hypercube) comprising two spatial and one spectral dimension. Further application of spectral unmixing procedure to every spatial pixel of the acquired hypercube allowed for calculation of abundances in a PL mixture component. Herein, we applied HSI to address multiplexing of nanoprobes for the in vivo SWIR PL bioimaging. We used two types of SWIR-emitting nanoparticles with emissions that overlap spectrally and cannot be easily distinguished in a conventional PL imaging with optical filters, despite their different spectral profile. Figure 1 illustrates the problem of the spectral mixture of PL from these nanoparticles and the way to overcome it using band sequential unmixing with HSI.

The HSI was applied to acquire the spectrally selective and sensitive PL imaging for both types of nanoparticles. To unmix the SWIR PL spectral profiles, the unmixing protocol was developed, allowing us to obtain quantitative and spatial mapping of components in the mixture, with determination of intensity distributions in addition to abundances. The SWIR HSI techniques and the developed unmixing protocol were further applied to the in vivo imaging of mice subcutaneously injected with nanoparticles to demonstrate the applicability of HSI to multiplex SWIR PL nanoprobes in the in vivo imaging.

## Methods

### Nanoformulation Preparation and Characterization

#### Synthesis of Polymeric Core-Shell Nanoparticles Loaded with Organic Fluorescent NIR-SWIR Dye

Polystyrene (PS)-poly-*N*-isopropylacrylamide (PNIPAM) core-shell nanoparticles were synthesized by microemulsion polymerization, with modification of the method described previously [44, 45]. First, PS-co-PNIPAM (10 wt.% of PNIPAM) core nanoparticles were prepared as follows. NIPAM (0.1 g), sodium dodecyl sulfate SDS (0.1 g), and 0.005 g of NaH_2_PO_4_ × H_2_O were dissolved in 45 ml of H_2_O. Styrene (1 g) was added dropwise at vigorous stirring when the temperature was increased to 60 °C. As the next step Ar was bubbled into the mixture for 30 min, the temperature was raised to 70 °C, and 0.08 g of K_2_S_2_O_8_ dissolved in 1 ml of H_2_O was injected to initiate the polymerization. Second, PNIPAM shell was layered onto the PS-co-PNIPAM core. For this purpose, the reactor was added aqueous solution of monomer NIPAM (1.8 g) and cross-linker *N*,*N*′-methylenebisacrylamide (BIS) (0.18 g) in 4 ml H_2_O using a syringe. The reaction was allowed to continue for 4 h at 70 °C. The mixture was cooled to room temperature and dialyzed during 74 h using cellulose membrane with MWCO 3500 Da. As a result, suspension of the PS-PNIPAM nanoparticles was fabricated; a core-shell structure of the nanoparticles is clearly revealed by the transmission electron microscopy (TEM) images (Fig. 3a). In order to obtain NIR-SWIR fluorescent PNPs, 2-butyl-6-[5-(2-butyl-1,3-dimethylcyclo-hepta[c]pyrrol-6(2H)-ylidene)penta-1,3-dien-1-yl]-1,3-dimethylcyclohepta[c]pyrrolium tetra-fluoroborate (labeled as JB9-08) fluorescent dye [46] was post-loaded [47] to PS-PNIPAM nanoparticles. Eight microliters of 1 mM JB9-08 dye solution in DMF was added to 2 mL of 0.25 wt% PNPs water suspension and kept for 24 h before use.

#### Synthesis of Core-Shell RENPs

RENPs were synthesized following the modified protocol reported elsewhere [48]. First, the α-NaYF_4_: 10%Yb^3+^, 30% Nd^3+^ core nanoparticles were prepared via decomposition of the metal trifluoroacetate at high temperature. In a typical procedure, 0.05 mmol Yb_2_O_3_, 0.15 mmol Nd_2_O_3,_ and 0.25 mmol Y_2_O_3_ were loaded into a 250 ml flask containing 5 ml deionized water and 5 ml TFA and heated to 90 °C for 1 h to yield a clear solution. The resulting clear solution was evaporated at this temperature under argon purge to get muddy powdered RE(TFA)_3_. Subsequently, 8 ml OA, 8 ml OM, 12 ml ODE, and 2 mmol NaTFA were added into the flask. The solution was heated to 120 °C and kept at that temperature for 30 min, followed by heating up to 300 °C for 30 min before naturally cooling down to room temperature. An argon environment was as applied during the whole synthesis process. The resulting nanoparticles were precipitated by adding 20 mL ethanol to the cooled reaction flask. After centrifugal washing with ethanol for three times, the collected white powder was finally dispersed in 10 ml hexane for further uses.

Second, the α-NaYF_4_: 10%Yb^3+^, 30% Nd^3+^@CaF_2_ core-shell RENPs were prepared via a seed-mediated epitaxial growth process, involving the use of α-NaYF_4_: 10% Yb^3+^, x% Nd^3+^ core as the seed and the corresponding growth in the shell precursor solution. To prepare the shell precursor, firstly, 2 mmol CaO with 5 ml deionized water and 5 ml TFA were added to a 250-ml flask and heated at 90 °C for 1 h to produce a clear solution. This solution was then evaporated at this temperature to yield the shell precursor of calcium trifluoroacetate (Ca(TFA)_2_). Next, 0.5 mmol NaYF_4_: 10% Yb^3+^, 30% Nd^3+^core nanoparticles, 7 ml OA, and 7 ml ODE were all added to the flask. The solution was then heated to 120 °C for 30 min, followed by heating up to 300 °C for 60 min before naturally cooling down. The whole process was carried out under an argon environment. The resulting core-shell nanoparticles were precipitated by adding 20 mL ethanol to the cooled reaction flask. After centrifugal washing with ethanol for three times, the collected core-shell NPs were finally dispersed in 10 ml hexane for further uses. For preparation of the aqueous dispersion, the prepared α-NaYF_4_: 10%Yb^3+^, 30% Nd^3+^@CaF_2_ core-shell RENPs (5 mL hexane dispersion) were firstly mixed with 5 mL *N*,*N*-Dimethylformamide (DMF) solution of nitrosonium tetra-fluoroborate (NOBF_4_) (0.1 M) at room temperature. A gentle shaking was applied to the mixture till an observation of RENP precipitation. Subsequently, toluene and hexane (1:1, volume) were added into the mixture, which was then centrifuged at 10000 rpm for 10 min. The precipitate was collected and dispersed in 5 mL DMF. Secondly, 250 mg poly(acrylic acid) (PAA, MW = 18,000) was added to the 5 mL DMF solution of NOBF_4_-treated RENPs, which was heated to 80 °C and kept at this temperature for 30 min under vigorous stirring. After that, the NPs were precipitated by adding acetone, washed with ethanol, and finally dispersed in distilled water.

#### Transmission Electron Microscopy

The morphologies of PNPs and RENPs were assessed using transmission electron microscopy (TEM). To be imaged with TEM, 10 μL of nanoparticle suspension was dropped onto the carbon support films stabilized with formvar. In order to visualize core-shell structure of PNPs, they were negatively stained with 1% water solution of phosphotungstic acid prior to dropping onto the support films. The carbon support films were air-dried and washed with 5 μL of pure water. The images were obtained by operating at an acceleration voltage of 100 kV on TEM (JEM-1230, JEOL).

#### Photoluminescence Spectroscopy

PL spectra for both types of nanoparticles were measured in NIR and SWIR ranges using a Fluorolog-3 spectrofluorometer equipped with iHR320 spectrometer for NIR-SWIR range (Horiba); a fiber-coupled laser diode emitting at 808 nm (QSP-808-4, QPhotonics) was used to excite PL from PNPs and RENPs.

### Hyperspectral Imaging System

**Fig. 2:**
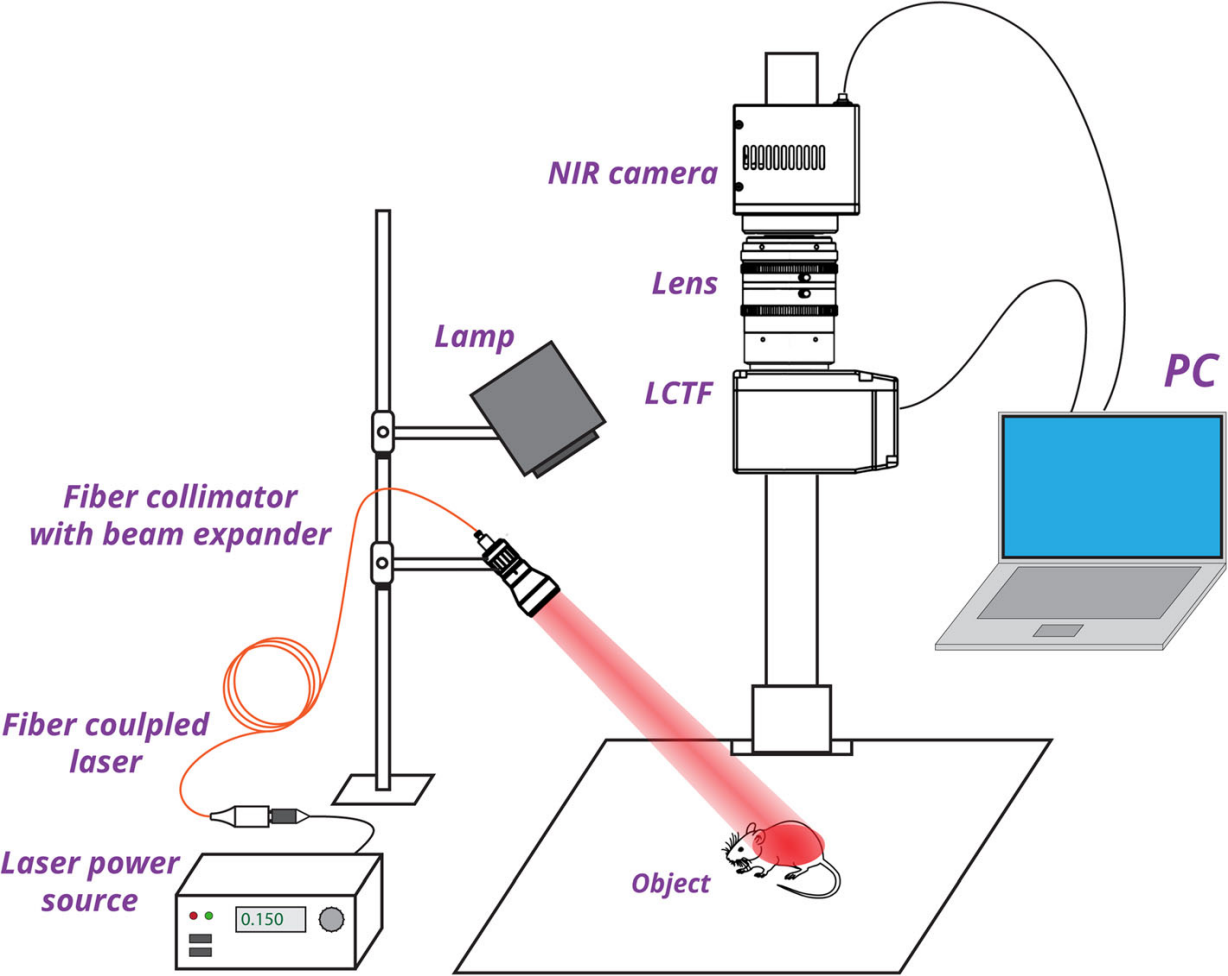
Schematic diagram of the NIR-SWIR hyperspectral imaging system

Homebuilt SWIR HSI system exploits band sequential acquisition method (Fig. 2) and includes NIR camera (Xeva-1.7-320, Xenics, Belgium), focusing optics (TEC-M55MPW, Computar, USA), and liquid crystal tunable filter (Varispec LNIR 20-HC-20, PerkinElmer, USA) as a dispersive element. The system has 340 × 258 pixel resolution and operates in 900–1700 nm spectral range. Illumination sources in the system include incandescent lamp (for image alignment, focusing, and bright field imaging) and 808-nm fiber-coupled laser diode (QSP-808-4, QPhotonics, USA), powered with laser power source (Laser Source 4308, Arroyo Instruments, USA), for PL excitation in PL imaging. Spectral data cube acquisition was performed by sequential tuning of LCTF transmittance of 20-nm spectral width in the range from 900 to 1200 nm with 10-nm step and capturing corresponding images. Exposure time of NIR camera during HSI acquisition was set to 200 ms. Laser power density on the sample was fixed at ~ 100 mW/cm^2^. For acquisition of PL images in whole NIR-SWIR range, LCTF was replaced by 850-nm-long pass filter (Edmund Optics, USA).

### Spectral Unmixing Software

For analysis of obtained spectral data cube, we developed spectral unmixing algorithm using the MATLAB environment. The spectrum of every pixel is considered as a linear mixture of known endmembers: $F\left(\lambda \right)=\sum \limits_i{a}_i{G}_i\left(\lambda \right)$, where *F*(*λ*)—pixel spectrum, *G*_*i*_*—*endmembers spectra, and *a*_i_ are abundances of endmembers. The purpose of the unmixing software is to estimate the abundances of known endmembers by solving linear spectral mixture analysis (LSMA) problem in every pixel of acquired spectral data cube. Suppose, *L* is number of spectral bands, and *p* is number of endmembers present in a mixture. Then, LSMA problem can be stated as ***F*** = ***Ga*** + ***n***, where ***F*** is *L* × 1 vector of pixel intensities, ***G*** is *L* × *p* matrix containing all endmembers vectors, ***a*** is *p* × 1 vector of unknown abundances, and ***n*** is *L* × 1 error vector. Least squares error (LSE)-based method to solve LSMA problem can be formed as following optimization task: min_***a***_{(***F*** − ***Ga***)^*T*^(***F*** − ***Ga***)}, and the classical solution is ***a***(***r***) = (***G***^*T*^***G***)^−1^***G***^*T*^***F***. However, such solution may contain negative values for abundances, which have no physical meaning. To resolve this obstacle, LSE optimization task has to be modified: min_***a***_{(***F*** − ***Ga***)^*T*^(***F*** − ***Ga***)}, subject to ***a*** ≥ 0. To solve this task, we utilize iterative algorithm, based on non-negativity constrained linear spectral mixture analysis (NC-LSMA) that is described in details in [49, 50]. After calculation of abundances for every component, they are mapped as 2D colormap graphs. Finally, unmixing software yields intensity images of the corresponding components, based on obtained abundances: ${I}_i\left(x,y\right)={a}_i\left(x,y\right)\sum \limits_{\lambda }{G}_i\left(\lambda \right)$, where *I*_i_(*x*, *y*)—integral intensity of *i*-th endmember in pixel, and *a*_*i*_(*x*, *y*)—*i*-th abundance.

### Animal Experiments

The BALB/c nude mice (Source: The Jackson Laboratory, USA) were bred under dark and aseptic conditions in a small animal facility. All animal experiments were conducted in compliance with the criteria of the National Regulation of China for Care and Use of Laboratory Animals. Prior to imaging, male nude mice (6 weeks old, 20 ± 2 g) were anesthetized with 5% chloral hydrate (0.06 ml per gram of mouse weight) by intraperitoneal injection. For SWIR PL imaging in vivo, nanoparticles were suspended in 10x phosphate buffered saline (PBS) and 100 μL of each PBS suspension of PNPs, RENPs, or their mixture were subcutaneously injected into the mice.

## Results and discussion

**Fig. 3:**
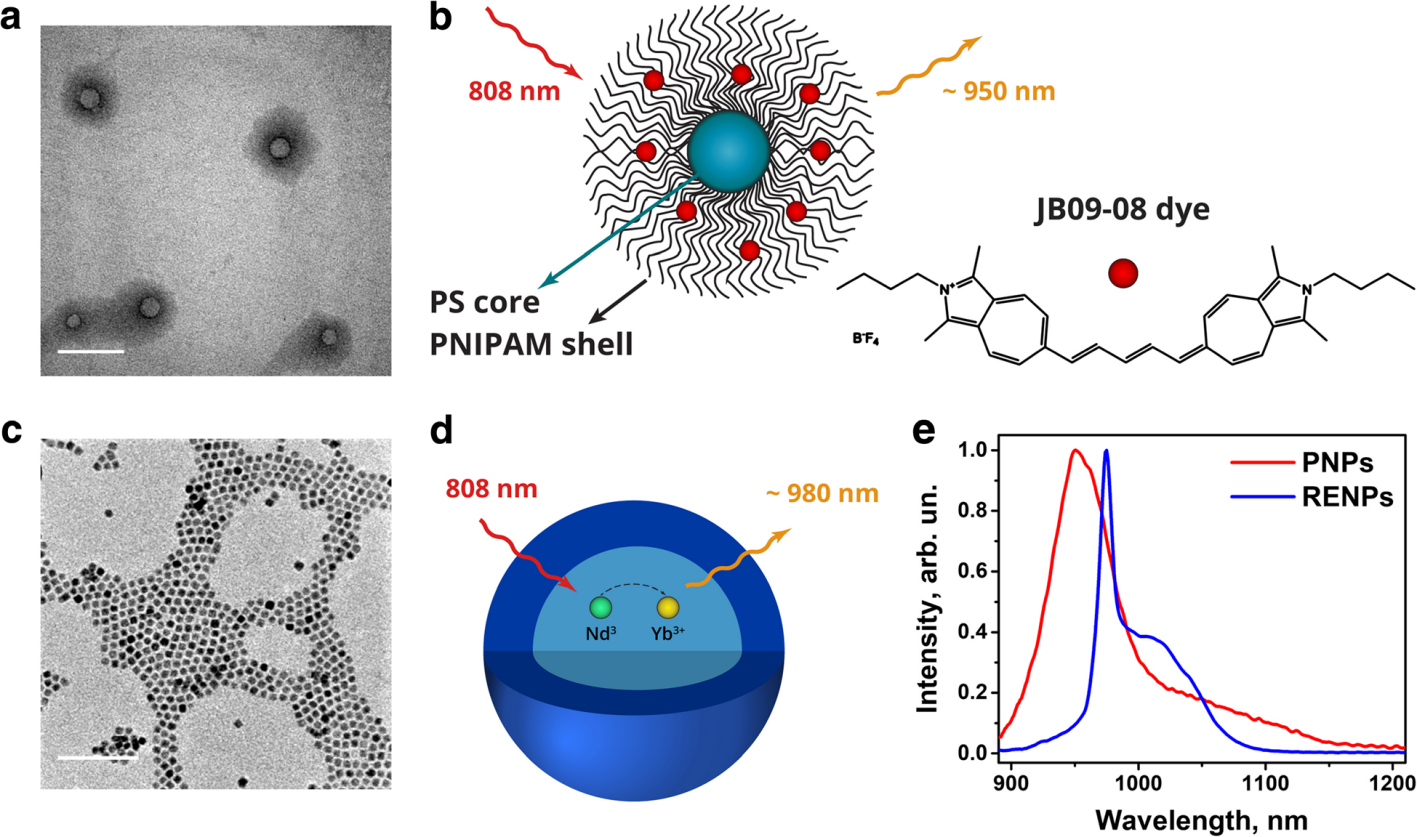
Characterization of PNPs and RENPs. TEM image (**a**) and schematic structure (**b**) of PNPs loaded with JB9-08 dye. TEM image (**c**) and schematic structure (**d**) of RENPs. **e** Normalized PL emission spectra of PNPs-JB9-08 and RENPs suspensions under 808 nm excitation. Scale bars, 100 nm

Two types of SWIR-photoluminescent NPs were prepared for use in HSI: (1) PNPs post-loaded with JB9-08 dye, which is virtually non-fluorescent in aqueous solutions [46] but, as shown by us [51], restores its fluorescence in water through post-loading into polymeric matrix of PNPs (Fig. 3a and b) and (2) PAA-coated NaYF_4_:10%Yb^3+^,30%Nd^3+^@CaF_2_ core-shell rare-earth ion-doped nanoparticles (RENPs, Fig. 3c and d). Nd^3+^ ions in the core of RENPs can be excited with ~ 808 nm light (^4^I_9/2_ → ^4^F_5/2_ transition) and transfer energy to Yb^3+^ ions, that emit in 950–1100 nm range with peak at ~ 975 nm (^2^F_5/2_ → ^2^F_7/2_ transition). RENPs core was coated with inert CaF_2_ shell to reduce nonradiative losses through surface defects and interaction with surrounding environment [48]. Both nanoformulations exhibit photoluminescence in 900–1200 range under 808 nm excitation (Fig. 3e).

**Fig. 4:**
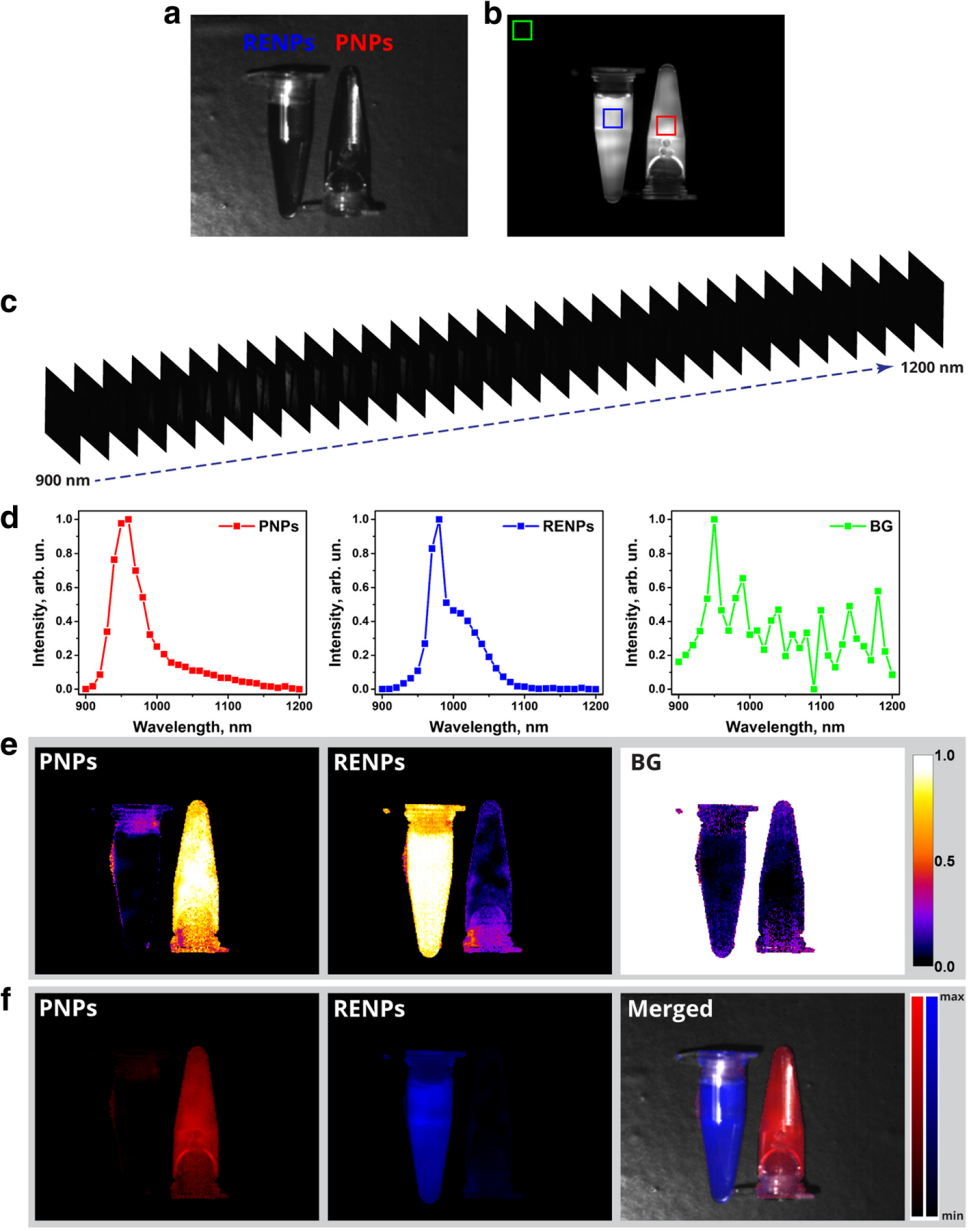
HSI of microcentrifuge tubes containing RENPs and PNPs. **a** Bright field image of microcentrifuge tubes with PNPs and RENPs suspended in PBS. **b** PL image of PNPs and RENPs samples excited with 808 nm (850-nm-long pass filter was used for image acquisition). **c** Scheme illustrating hypercube composed of HSI frames. **d** Spectral profiles of PNPs, RENPs, and background (BG) averaged from ROI shown in **b** as red, blue, and green squares, correspondingly. **e** Color maps of components abundances. **f** Reconstructed intensity images of the PL components and PL/bright field merged image

It should be noted that PL emission spectra of the nanoparticles obtained with spectrofluorometer cannot be used as endmembers in spectral unmixing software because of two reasons. First, the sensitivity of HSI system is not spectrally corrected, unlike in conventional spectrofluorometer, so the acquired emission spectral profile is different for two acquisition methods. Second, HSI frames are collected with 10 nm step, although the spectral bandwidth of LCTF is 20 nm. That results in the signal overlapping in neighboring frames, causing distortion of obtained spectral profile in comparison with the one measured with spectrofluorometer. To overcome these obstacles, endmembers (spectral profiles of PNPs and RENPs samples) were acquired with HSI system. With this aim, both types of nanoprobes were suspended in PBS and their PBS suspensions were placed in microcentrifuge tubes (Fig. 4a). It is hardly possible to differentiate two samples with conventional PL imaging, as their PL emission spectra overlap (Figs. 4b and 3e). Using HSI system, 31 PL images were collected in a spectral range from 900 to 1200 nm with 10-nm step (Fig.4c). Spectral profiles of PNPs, RENPs, and background (BG) were calculated through the spectral dimension of the spectral data cube by averaging signal in areas marked by red, blue, and green squares, correspondingly (Fig.4b). The obtained spectral profiles for PNPs and RENPs (Fig. 4d) were found to be similar to ones measured by spectrofluorometer and were later used as endmembers for the spectral unmixing. Thereafter, with PNPs, RENPs, and BG spectral profiles as endmembers, corresponding component abundances are calculated and mapped as 2D colormap plots (Fig. 4e). Abundances were calculated for every spatial pixel of the hypercube and were represented as a value from 0 to 1, with 0 and 1 indicating the full absence and full abundance of the component, respectively. Sum of abundances of all the components in every pixel is equal to 1. It should be noted that unmixing software used thresholding by intensity of the signal to eliminate errors caused by low-intensity pixels. If maximal intensity of a pixel along spectral dimension was less than 5% of the maximum of whole hypercube, such a pixel was considered to be fully abundant with noise component. Furthermore, integral intensity images of PNPs and RENPs were calculated considering corresponding abundances and eliminating background component (Fig. 4f). As it was expected, both abundance mapping and integral intensity images demonstrate full abundance of PNPs in right tube and RENPs in left tube, with slight error caused by the PL emission scattering and imperfection of the unmixing algorithm.

**Fig. 5:**
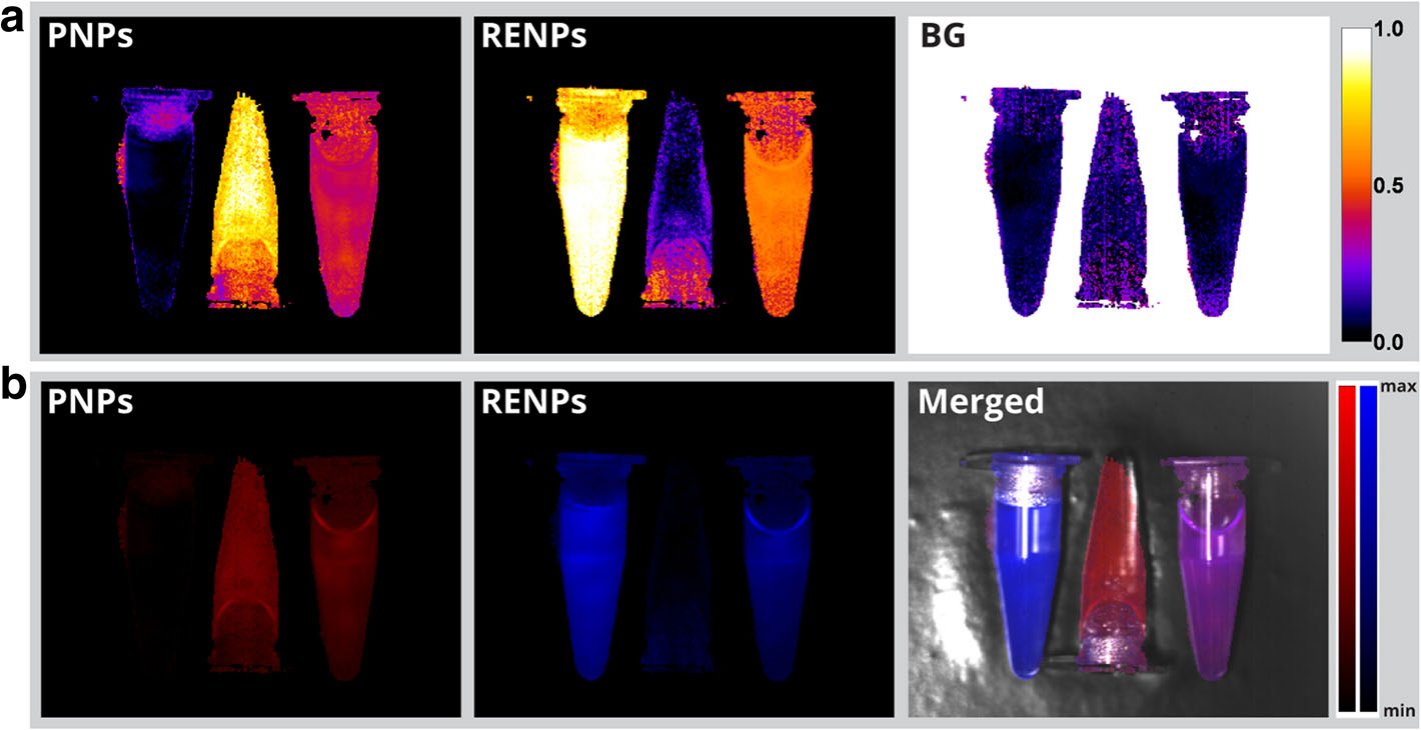
HSI of microcentrifuge tubes containing (left to right) RENPs, PNPs, and mixture of both. **a** Abundances of PNPs, RENPs, and background (BG). **b** Reconstructed PL intensity images and PL/bright field merged image

Spectral unmixing is also able to distinguish the mixture of PNPs, RENPs. To demonstrate this, three microcentrifuge tubes were filled with solutions of PNPs, RENPs, and their mixture. After acquisition of hypercube and spectral unmixing procedure, abundance mapping indicates full presence of PNPs in the first microcentrifuge tube, RENPs in the second, and a mixture of the two in the third one (Fig. 5a). Reconstruction of intensity images was further performed to reveal intensity distribution of the PNPs and RENPs PL signals in the specimen (Fig. 5b).

**Fig. 6:**
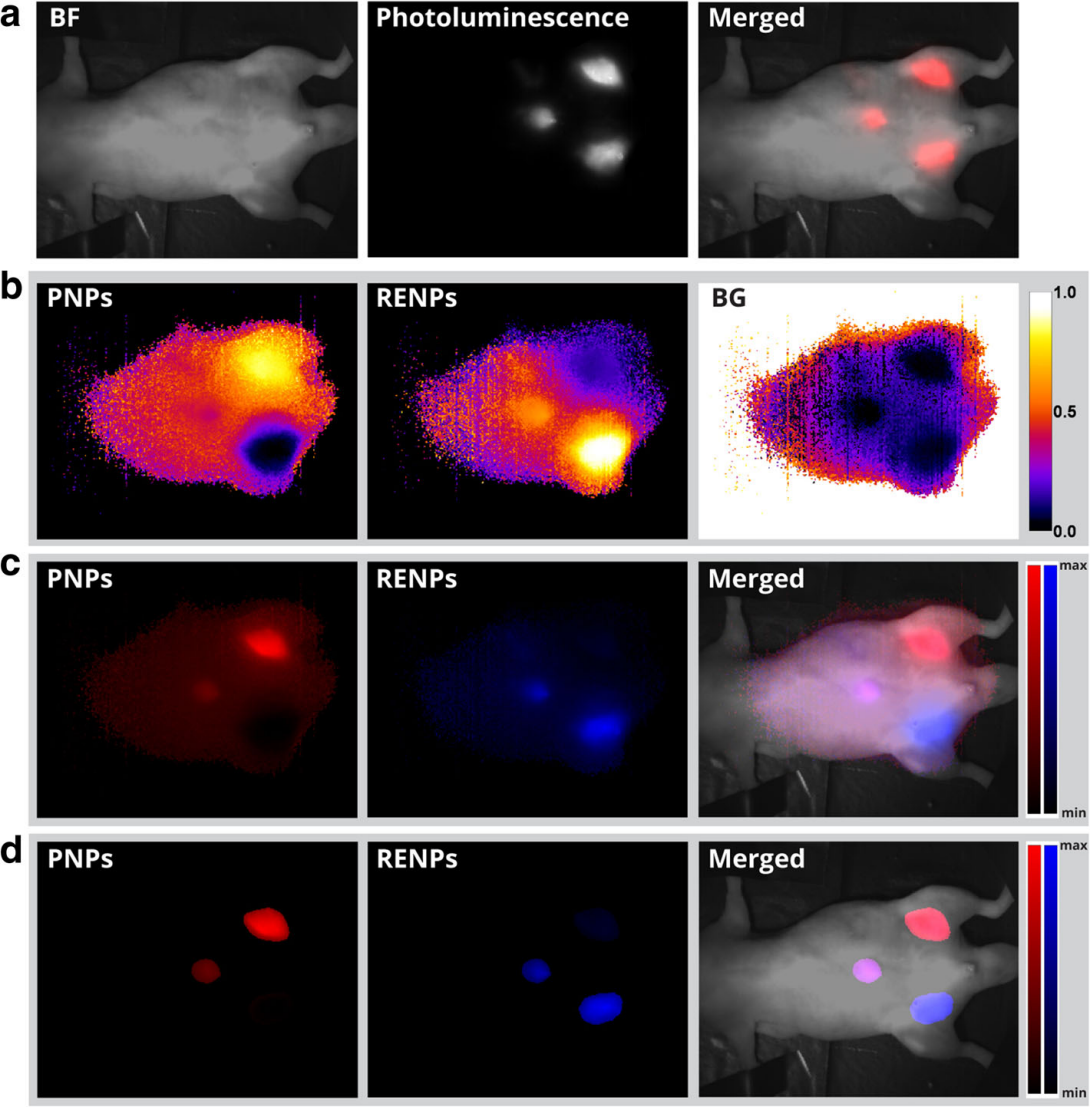
HSI of mouse subcutaneously injected with PNPs (top right injection site), RENPs (bottom right), and RENPs/PNPs mixture (left). **a** Bright field (SWIR), SWIR PL, and merged images acquired with 850-nm-long pass emission filter. **b** Abundances of PNPs, RENPs, and background (BG). **c** Corresponding reconstructed intensity images. **d** PL intensity images reconstructed from abundances with threshold level of 15% and PL/bright field merged image.

We have further demonstrated the applicability of our HIS method to multiplex the spectrally overlapped nanoprobes in SWIR PL imaging in vivo. Nude mouse was anesthetized and subcutaneously injected with PNPs, RENPs, and a mixture of the two. NIR imaging with 850-nm-long pass filter shows three indistinguishable photoluminescent spots (Fig. 6a). After performing HSI and analysis, abundances mapping shows full abundances of PNPs and RENPs in the top and bottom injection sites, respectively, while mixture of two was identified in the left injection site (Fig. 6b). In addition, to acquire the PL intensity distribution, intensity reconstruction was performed (Fig. 6c). By adjusting thresholding value in the unmixing software to 15% (estimated empirically), it became possible to remove noise in areas adjacent to emissions spots (Fig. 6d). Such results indicate that unlike the conventional PL imaging modality, which employs long or bandpass optical filters, HSI not only is able to map intensity distribution in the specimen, but also can multiplex the components present in the mixture by identifying the spectral profile of intensity in every pixel of the image.

Thus, the combination of HSI acquisition and spectral unmixing processing was applied in this work to obtain a multiplexed imaging of the spectrally overlapping SWIR PL nanoprobes. A few issues should, however, be considered in regard to the applicability of HSI modality. First, due to the spectrally narrow acquisition, every HSI frame deals with relatively low signal intensity (especially in the case of the spectrally broad PL emission form the organic moieties). In addition, an increase in the PL exciting laser power in biological imaging is limited by danger of tissue damage (or phototoxicity). This results in higher requirements for the brightness of the PL probes and their photostability, as HSI can require order of magnitude longer acquisition time in comparison with conventional PL imaging. It should also be noted that the linear mixture analysis and spectral unmixing algorithm can work satisfactorily for multiplexed PL imaging of biological tissues in the spectral range where tissue transmittance does not change much. In contrast, if tissue transmittance has distinctive features in the spectral range of HSI, linear spectral mixture model may be hardly applicable (especially in case of deeper tissues) and nonlinear models can be considered.

## Conclusions

In conclusion, we developed a hyperspectral SWIR bioimaging technique and applied it for multiplexing of nanoparticles emitting in SWIR spectral range. Two types of nanoparticles with overlapping PL spectra, which are undistinguishable in conventional imaging, were successfully multiplexed by their PL spectral profiles using hyperspectral acquisition along with the linear spectral mixture analysis algorithm. The developed method was successfully employed for multiplexed imaging of SWIR PL nanoparticles both in sample suspensions and injected in small animals. With SWIR bioimaging having superior resolution at higher imaging depth, SWIR HSI approach holds a great potential for use in various applications requiring multiplexed imaging with NIR-SWIR PL nanoprobes. Furthermore, as SWIR bioimaging is at a very early stage, there is plenty of room for the development of biomedical applications of PL probes in a combination with HSI.

## Data Availability

All data generated or analyzed during this study are included in this published article.

## Abbreviations

BG: Background
HSI: Hyperspectral imaging
LCTF: Liquid crystal tunable filter
NIR: Near-infrared
NPs: Nanoparticles
PL: Photoluminescence
PNIPAM: Poly-*N*-isopropylacrylamide
PNPs: Polymeric nanoparticles
PS: Polystyrene
RENPs: Rare-earth doped fluoride nanoparticles
SWIR: Short-wave infrared
TEM: Transmission electron microscopy
